# Somatic Embryogenesis of Selected *Pinus* Species: Developmental Stages of *Pinus peuce* and *Pinus heldreichii*

**DOI:** 10.3390/plants15030411

**Published:** 2026-01-29

**Authors:** Dragana Stojičić, Vlado Čokeša, Marija Marković, Olga Radulović, Branka Uzelac

**Affiliations:** 1 Department of Biology and Ecology, Faculty of Sciences and Mathematics, University of Niš, Višegradska 33, 18000 Niš, Serbia; 2 Institute of Forestry, Kneza Višeslava 3, 11000 Belgrade, Serbia; vlado.cokesa@gmail.com; 3 Department of Plant Physiology, Institute for Biological Research “Siniša Stanković”—National Institute of the Republic of Serbia, University of Belgrade, Bulevar Despota Stefana 142, 11108 Belgrade, Serbia; marija.nikolic@ibiss.bg.ac.rs (M.M.); olga.radulovic@ibiss.bg.ac.rs (O.R.); branka@ibiss.bg.ac.rs (B.U.)

**Keywords:** clonal propagation, Macedonian pine, Bosnian pine, megagametophyte, tissue culture, forest conservation

## Abstract

Somatic embryogenesis (SE) represents the most efficient and scalable technology for the mass clonal propagation and genetic improvement of superior conifer genotypes, which is crucial for meeting global wood demand and supporting forest adaptation to climate change. Despite its immense potential, SE in the genus *Pinus* still faces major limitations, including low initiation frequencies, restricted explant availability, and pronounced genotype dependence. This review synthesizes current knowledge on the factors influencing SE in *Pinus* species, with a specific focus on two ecologically vital Tertiary relicts endemic to the Balkan Peninsula: *Pinus peuce* (Macedonian pine) and *Pinus heldreichii* (Bosnian pine). For these species, traditional vegetative propagation methods are difficult or ineffective, making SE the priority approach for clonal propagation. Detailed studies on these species revealed that SE induction is highly dependent on the explant type and developmental stage. Successful embryogenic tissue formation was achieved only from whole megagametophytes containing immature zygotic embryos, within a narrow developmental window spanning 4–10 weeks post-fertilization. Furthermore, medium composition, particularly reduced ammonium concentration, proved critical for *P. heldreichii* success. These findings underscore the need for continued, species-specific optimization to overcome current bottlenecks and realize the full potential of SE for the conservation and sustainable clonal forestry of these high-value pines.

## 1. Introduction

Propagation of pines and other economically important forest tree species has traditionally relied on seeds, which are relatively easy to collect, store, and sow [1]. However, seed-based propagation is limited by inconsistent seed quality due to periodic cone harvests [2], low germination rates, seed dormancy [3,4,5,6], and genetic heterogeneity from sexual recombination, preventing the capture of superior parental genotypes [1]. Conventional vegetative methods (e.g., cuttings, layering, grafting) produce genetically identical plants but are severely constrained in mature conifers due to poor rooting success influenced by donor age and genotype [7], as well as the long generation times and extended juvenile-to-mature transitions typical of pines [8].

In vitro propagation techniques offer alternatives, but different organogenesis approaches [9,10,11,12,13,14,15,16,17] remain bottlenecked by difficult adventitious rooting, limiting scalability for clonal forestry [13,18]. In contrast, somatic embryogenesis (SE) offers distinct advantages by producing bipolar structures that develop directly into complete plantlets without a separate rooting phase [19]. SE is thus the most scalable technology for mass clonal propagation of elite conifers with traits such as superior growth, wood quality, and stress resistance [19,20,21]. This is increasingly vital as rising global demand for wood products—driven by population and economic growth—cannot be met sustainably by natural forests alone. Planted forests already supply a substantial and growing share of industrial roundwood, with projections emphasizing the need for intensified production from high-performing plantations in the coming decades [22]. Clonal propagation through SE accelerates breeding and deployment of adapted genotypes, supporting sustainable forestry goals [1].

However, SE in many conifer species and particularly in the genus *Pinus*, still faces major technical hurdles, including low initiation frequencies, recalcitrant seed sources, poor culture survival, and premature embryo maturation [20]. These limitations result in slow initial growth, low germination rates and elevated production costs for somatic seedlings derived from successfully initiated genotypes, which have historically restricted SE to juvenile explants [23]. Despite these obstacles, recent progress in understanding the underlying molecular and physiological mechanisms of in vitro plant regeneration has led to markedly improved SE protocols [24]. Building on these mechanistic insights, researchers have refined induction media and explant selection to boost embryogenic callus formation and proliferation, which has expanded SE to new or recalcitrant species, with first-time successes reported [25,26,27]. Recent work has dramatically increased mature embryo yields through combined liquid-solid induction methods, hormone pretreatments, and additives, along with improved germination rates [27,28]. Cryopreservation protocols have been developed to preserve embryogenic lines long-term without losing embryogenic potential, outperforming repeated subculturing, which often leads to decline [26,28].

This is especially relevant for Tertiary relict pine species of high conservation value, such as *Pinus peuce* (Macedonian pine) and *Pinus heldreichii* (Bosnian pine) in the Balkan Peninsula. Macedonian pine is endemic to the Balkans and belongs to the subgenus *Strobus* (Haploxylon), whereas Bosnian pine is sub-endemic to the Balkans and southern Italy and is classified in the subgenus *Pinus* (Diploxylon) [29]. Historically, these species formed extensive dominant forests across the region, but intensive anthropogenic pressure has reduced their distribution to scattered, fragmented remnants on Mediterranean and sub-Mediterranean mountain ranges [30].

Both species occupy altitudinal ranges of 800–2400 m and are well adapted to the short growing season, tolerating hot dry summers with intense solar radiation and severe winters [31]. Bosnian pine predominates on permeable limestone substrates, particularly steep dry slopes, while Macedonian pine prevails in wetter sites; mixed stands are common on silicate substrates in areas previously logged or heavily thinned, where increased light favors the heliophytic *P. heldreichii* [32,33]. Despite these ecological differences, the reproductive strategies of the two species share important constraints. Seeds of *P. peuce* and *P. heldreichii* exhibit dormancy, requiring cold stratification (approximately 6 months for *P. peuce* and 6 weeks for *P. heldreichii*) for successful germination [3], with highly variable production among years, individual trees, and even cones on the same tree [34,35,36]. Yet, despite their slow growth and irregular seed production, both pines are highly valuable for afforestation due to exceptional tolerance of low temperatures, extreme drought and air pollution, making them key for restoring high-mountain ecosystems amid climate change [32]. Given their limited distributions, both species require targeted conservation measures and special protection [30].

Certain research approaches developed for widely distributed, commercially important conifers have proved not directly applicable to Balkan endemics *P. peuce* and *P. heldreichii*. This arises from key differences in genetic background, population structure, ecological adaptations, and life-history traits that underscore the uniqueness of these Tertiary relict species. *P. peuce* and *P. heldreichii* have low genetic diversity from glacial refugia survival, with small, fragmented populations causing strong differentiation even among nearby stands, in contrast widespread species feature high gene flow, broader genetic bases, and support for rapid breeding. In essence, their narrow endemic distribution, refugial isolation, low variability, and extreme montane adaptations make *P. peuce* and *P. heldreichii* uniquely vulnerable and distinct.

Developing robust, cost-effective SE methods therefore remains crucial not only for commercial pines but also for these relict species, to enable large-scale multiplication and field-deployment of elite clones for reforestation, conservation, and industrial needs. In this review, we summarize the current knowledge and key challenges associated with SE in *P. peuce* and *P. heldreichii*, highlighting their potential to overcome the remaining bottlenecks in conifer forestry.

## 2. Somatic Embryogenesis in Conifers

SE is a developmental process in which somatic cells undergo the characteristic morphological and physiological stages of embryonic development without prior gamete fusion, ultimately forming a complete plant. In vitro, diploid somatic cells from vegetative tissues or haploid microspores develop into bipolar embryos through a sequence of stages analogous to those of zygotic embryogenesis (ZE) in vivo [19]. Unlike ZE, where the resulting embryo has a unique recombined genome distinct from the parent plant, SE produces embryos that are genetically identical to the donor plant [37]. Beyond these genetic differences, the two processes also differ markedly in their developmental environments: zygotic embryos (ZEs) develop inside the protective seed and receive an optimal combination of precisely timed nutrients and growth regulators supplied by the surrounding megagametophyte tissue, whereas somatic embryos (SEs) are cultured on artificial nutrient media and their development is therefore highly dependent on medium composition [20]. Nutritional requirements vary considerably among species, so the induction and proliferation of SEs still largely rely on empirical, trial-and-error optimization [38]. Nevertheless, numerous studies have demonstrated that SEs from mature conifers, produced via plant tissue culture, can reach morphological and physiological quality comparable to that of mature ZEs, making SE a powerful tool both for studying embryo developmental physiology and for biotechnological applications in clonal forestry [39,40,41].

SE is a powerful tool for the mass propagation of elite conifer genotypes, for the production of synthetic seeds, and as a platform for genetic engineering and the regeneration of genetically modified forest trees [39,42,43,44]. Despite the availability of other clonal propagation methods for forest trees, SE is increasingly accepted as the method of choice for rapid vegetative multiplication of superior conifer genotypes, especially when conventional techniques are inadequate or too slow [45,46]. In many conifer species, however, reliable clonal propagation via SE remains challenging, as it is still largely restricted to juvenile explants [23]. This limitation arises from maturation-associated recalcitrance, during which older tissues lose the ability to undergo the profound biochemical and epigenetic reprogramming required for embryogenic induction [47]. Consequently, a major ongoing challenge is the development of standardized, genotype-independent protocols that can be applied across the diverse conifer species and families used in operational forestry.

### 2.1. Historical Perspective and Development of SE in Pinus *spp.*

The family Pinaceae is the largest and most economically important conifer family, comprising approximately 232 species in 11 genera (*Abies*, *Cathaya*, *Cedrus*, *Keteleeria*, *Larix*, *Nothotsuga*, *Picea*, *Pinus*, *Pseudolarix*, *Pseudotsuga*, and *Tsuga*). Among these, *Pinus* is by far the largest genus, with 111 currently recognized species [29,48]. The genus is predominantly distributed throughout the Northern Hemisphere with the notable exception of *Pinus merkusii*, which occurs in Indonesia, the Philippines, Myanmar, Thailand, Laos, Cambodia, and Vietnam [49,50]. Numerous *Pinus* species have also been widely introduced to the Southern Hemisphere for commercial forestry, notable examples being *Pinus pinaster* in Australia, New Zealand, Argentina [51], *Pinus pinea* in Chile [52], *Pinus elliottii* in Australia, South America [53], *Pinus radiata* in New Zealand, Chile and Australia [54], *Pinus patula* in South Africa [55].

Pines are ecologically important, often forming dominant or co-dominant components of boreal, subalpine, temperate and tropical forests. Economically, they represent a major source of timber, pulp and paper, resins, charcoal, and ornamental products [56], as well as bioenergy, natural oils, and food, particularly edible seeds [57]. Given their ecological and commercial significance, the development of a reliable micropropagation system capable of producing genetically transformed plants is highly desirable. In this regard, regeneration via SE offers the greatest potential for large-scale clonal propagation and genetic improvement [58], while simultaneously providing a powerful in vitro model for studying embryogenesis, cell differentiation and totipotency in conifers [59].

SE was first achieved in carrot root cultures more than six decades ago [60,61], and since then interest in using this process for the propagation of diverse plant species has grown steadily. Consequently, SE has been successfully induced in numerous plant species across virtually all major taxonomic groups, with particular success in agricultural and horticultural crops [62,63], forest trees, and other economically [64,65,66,67] or ecologically important species [68,69,70].

The fact that cells from a broad range of plant species can undergo SE under appropriate in vitro conditions indicates that the process is fundamentally universal, although the specific requirements remain strongly species- and often genotype-dependent. It is widely believed that SE is achievable in all plant species provided the right explant, culture medium, and environmental conditions are identified [19]. Successful induction and development of SEs therefore require a precisely formulated nutrient medium containing optimal concentrations and ratios of macronutrients, micronutrients, carbon sources, vitamins and growth regulators, under tightly controlled culture conditions [71,72].

Initial attempts to induce SE in gymnosperms date back to the late 1970s and early 1980s, when filamentous polar structures resembling early pine embryogenic stages were obtained in *Pinus banksiana* [73]. Real progress came in the mid-1980s, with the first regeneration of complete SEs from immature embryos of *Picea abies* [74,75] and from megagametophyte of *Larix decidua* [76]. However, the structural and physiological complexity of woody perennials, and conifers in particular, has resulted in considerably slower progress compared with herbaceous species [77]. High genetic variability within species and pronounced heterogeneity among genera still hinder direct transfer of protocols between taxa [78]. Over the past four decades, continuous technical improvements have nevertheless markedly increased somatic embryo induction and development in conifers. To date, SE has been reported in approximately one-fifth of the more than 110 recognized *Pinus* species [79,80].

Since the first successful induction of SE in conifers, research has focused on optimizing in vitro culture conditions to improve both initiation frequency and embryo quality [81]. The first critical step is the selection of suitable explants, followed by optimization of the culture medium composition and tightly controlled environmental conditions [71,81]. Each of these factors plays an essential role in the induction of embryogenic tissue and in all subsequent stages of somatic embryo development.

### 2.2. Selection of Explants

The selection of suitable explants is a critical determinant for successful induction of SE. In a wide range of plant species, explants that have been successfully employed include immature and mature ZEs, immature ovules, seedlings, hypocotyl, cotyledons, apical meristems, root parts, flower parts, seed embryos, endosperm, anthers and microspores [72]. Although virtually all plant cells contain a complete set of genes with potential to guide all developmental stages, only certain cell types—typically meristematic or partially differentiated cells—are competent to initiate the embryogenic program [82]. In conifers, explant choice is further complicated by the frequent need to target specific superior genotypes identified in mature trees. However, tissues from adult trees are highly differentiated and physiologically conditioned, which makes the induction of SE particularly challenging. Moreover, both the type of tissue and its developmental stage strongly influence in vitro responsiveness [83].

In the genus *Picea*, SE has been successfully induced from a range of relatively mature explants, including shoot tips of seedlings [84,85], cotyledons [86], young needles [87], lateral buds [88], and mature ZEs [89].

Initially, researchers assumed that SE protocols developed for spruce could be directly applied to other conifers, including pines. However, it soon became evident that *Pinus* species require substantial modifications to these methods [90]. *Pinus* species generally respond best to juvenile explants, particularly embryos at the precotyledonary and cotyledonary stages and megagametophytes containing immature ZEs [91,92]. Initiation is typically possible only within a narrow developmental window—often lasting a few weeks—coinciding with cleavage polyembryony, the timing of which can vary annually [8]. The optimal induction period occurs after fertilization but before cotyledon formation [93]. This critical timing has enabled successful SE induction in species such as *Pinus lambertiana*, *P. palustris*, *P. pinaster*, *P. heldreichii* [58,92,94,95,96]. Conversely, in *Pinus strobus* [97], *Pinus caribaea* [98] and *P. radiata* [99], immature ZEs proved unsuitable for SE induction, whereas fertilized ovules were markedly more effective.

The induction of SE in *Pinus* spp. is also strongly affected by the origin of the seed populations [100]. Differences among seed populations in their capacity to initiate SE arise not only from year-to-year variation but also from differences in the physiological maturity of the seeds. Because conifers exhibit highly irregular flowering and seed-development patterns, seeds collected on the same date are not necessarily at the same physiological stage [101]. Even immature cotyledonary-stage embryos of *Pinus nigra*, *P. heldreichii* and *P. peuce* typically produce only non-embryogenic callus and dehydrate rapidly in culture, while initiation frequencies decline sharply as ZEs mature [92,102,103].

Although immature ZEs generally yield higher initiation frequencies [43], their practical use as explants is constrained by low initiation rates in many genotypes, a narrow collection window, and strong genotypic dependence. A major drawback of using immature ZEs as explants is their limited seasonal availability, which confines the initiation period to a narrow window. It is therefore desirable to extend the initiation window to more advanced developmental stages [104]. Many of these limitations can be overcome by using mature ZEs isolated from stored dry seeds, which are available year-round and exhibit considerably lower genotype dependence [105]. Successful SE induction from mature ZEs has been achieved in some *Pinus* species, including *Pinus sylvestris* [106], *P. strobus* [107], and *P. nigra* [59,108]. Although early attempts to induce SE from mature ZEs of *P. nigra* were unsuccessful [102,109], the same research group subsequently reported the first successful initiation and development of SEs from this explant type [59]. However, induction from mature ZEs has repeatedly failed in numerous other taxa, including *P. caribaea* [110], *P. heldreichii* and *P. peuce*, despite extensive testing of various pretreatments and culture media.

Ultimately, both immature and mature zygotic embryo explants share a major inherent drawback: the unknown genetic potential of the resulting plants. This limitation can be circumvented by initiating embryogenic cultures from vegetative tissues of elite, field-tested mature trees (e.g., secondary needles or apical shoots), thereby ensuring that regenerated plants are genetically identical to the selected superior donor and retain its desirable traits [111]. Notably, successful initiation and establishment of embryogenic cultures from apical shoots of mature *P. patula* trees representing three distinct genotypes have been reported [112].

#### 2.2.1. Induction Rate

The induction rate of embryogenic tissue in conifers varies considerably both among and within species. In *Pinus* spp., initiation frequencies are generally strongly influenced by species, genotype, explant type, developmental stage of the zygotic embryo, and culture conditions. Even when a wide range of plant growth regulator (PGR) concentrations and combinations, or different carbon sources, was tested, induction frequencies in most *Pinus* species remained relatively low. Reported rates include 0.2–1.9% for *P. caribaea* [98]; 1.61–4.91% for *P. palustris* [58]; 1.53–24.10% for *P. nigra* [59]; 3.52–33.33% for *Pinus koraiensis* [113].

Low initiation frequencies are common in open-pollinated seed sources and are largely explained by genetic variation among selected trees [45]. Differences in embryogenic tissue induction rates within the same species are common and are primarily attributable to genotypic variation in the starting material. When grown under identical conditions, some families or individual trees consistently produce far higher initiation rates than others. This strong genotype effect is common in plant SE and represents a major obstacle to industrial-scale conifer propagation and to the deployment of elite tree breeding programs. Widespread adoption of the technology will therefore depend on a better understanding of the regulatory factors involved and on improved screening methods for high-yielding SE cell lines [82].

The developmental stage of the zygotic embryo at the time of collection is another critical factor. This narrow developmental window is clearly illustrated in *P. strobus*, where the average initiation rate of SE was highest (38.0%) in cones collected on 5 July and declined sharply to 13.4% by 2 August, reflecting rapid loss of embryogenic competence as embryos progressed toward cotyledon differentiation [114]. In addition, Finer et al. [115] obtained embryogenic tissue from only 3% of isolated immature ZEs of *P. strobus*, but the frequency increased to 54% when the entire megagametophyte was used as the explant. In *P. caribaea,* Laine and David [98] recorded only 0.2–1.9% induction when intact embryos were cultured with surrounding endosperm, and no embryogenic tissue formed when embryos were excised from the endosperm. Interestingly, an opposite trend was seen in maritime pine (*P. pinaster*): Bercetche and Pâques [95] obtained 8% initiation with megagametophytes containing immature embryos, but the frequency rose to 15% when precotyledonary ZEs were isolated and cultured alone.

Initiation frequency of SE also exhibited marked inter-annual and inter-study variation, yet occurred on all media tested, including PGR-free medium [92,103]. In *P. nigra*, this variation was particularly pronounced: one study reported only 3.06% of 1108 explants producing protruding embryogenic tissue [59], while in an earlier study by the same authors, rates ranged from 1.53 to 24.1% [109]. In contrast, more consistent initiation frequencies were obtained for *P. peuce* (4.4–8.1%) and *P. heldreichii* (5.8–8.4%). Similar genotypic and developmental-stage effects have been reported in other species, with induction frequencies of approximately 10% in slash pine *(P. elliottii*; [116]) and reported frequency rates from 9% [117] to 53.4% [118] in loblolly pine (*Pinus taeda*), depending on the seed family. Overall, the low and highly variable initiation frequencies typically observed in *Pinus* highlight the strong combined influence of genotype and the precise physiological state of the explant at collection.

#### 2.2.2. Influence of Cone Storage Conditions on Initiation Frequency

Refrigeration of seed cones prior to embryo dissection is a common and effective practice in conifer SE. It extends the period during which seeds remain suitable for culture initiation and, in some cases, enhances the induction of embryogenic tissue [91].

Ideally, megagametophytes containing ZEs should be excised and placed on initiation medium as soon as possible after cone collection. However, intact cones can be safely stored at 4–5 °C for up to two months (and in many species even longer) without reducing the potential to initiate embryogenic cultures [119]. Low-temperature storage (4–5 °C) appears to slow precocious germination and maintain embryo physiological status, sometimes resulting in higher initiation frequencies compared with freshly collected cones [59,118].

### 2.3. Formulating a Nutrient Medium

The success of SE in *Pinus* species largely depends on the composition of the nutrient medium. Commonly used basal media for the induction of SE include MS [120], GD [121], LP [122], SH [123], and DCR [124] (Table 1). These media are frequently modified to meet the specific requirements of individual species or genotypes. For example, Sommer et al. [14] modified GD medium by reducing the ammonium concentration, while Becwar et al. [100] modified MS medium by replacing NH_4_NO_3_ with glutamine and reducing the KNO_3_ concentration.

Embryogenic tissue has been successfully induced in various *Pinus* species using different nutrient media that vary considerably in their ammonium and nitrate ion contents, as well as in other macro- and micronutrients. In *P. taeda*, SE has been initiated on full- or half-strength MS [43] and on DCR medium [117], but induction consistently failed on LP medium [125]. In contrast, *P. nigra* readily produced SEs on LP medium, which has a high ammonium concentration [102], as well as on DCR medium, which has a relatively low ammonium level [126]. SH medium supported embryogenic tissue induction in *P. caribaea* [98], whereas MS medium with nearly double the standard ammonium concentration was also effective in this species [110]. In *P. elliottii*, embryogenic cultures were successfully established on both LP [116,127] and DCR media [42,128]. These examples illustrate that in *Pinus* species, although medium composition is important, the genotype and developmental stage of the zygotic embryo often play an equally or more decisive role in successful induction of embryogenic tissue.

Among the tested basal media, DCR has proven the most universally effective for embryogenic callus induction across a wide range of *Pinus* species, including *P. strobus* [97,115], *P. taeda* [117], *P. patula* [45,55], *P. palustris* [58], *P. lambertiana* [94], *P. nigra* [104], *P. koraiensis* [79]. Its superiority is likely linked to the presence of calcium nitrate—a component absent from the commonly used MS, GD, LP, and SH media—and to its balanced nitrogen nutrition.

Nitrogen nutrition is one of the critical factors influencing plant growth and development. The form and ratio of nitrogen sources supplied strongly determine the effectiveness of different media in SE [129]. Several *Pinus* species respond better to hybrid or specially modified media. For instance, the highest induction rates in *P. heldreichii* were obtained with GDS medium—a GD formulation modified by Sommer et al. [14] that is characterized by a markedly reduced ammonium concentration [92,96]. Similarly, embryogenic callus induction in *P. caribaea* was successful using SH macronutrients combined with MS micronutrients and DCR vitamins [98]. In some cases, reducing salts or modifying nutrient media has also proven efficient for mitigating browning and necrosis of embryogenic tissue [58].

Efforts to further optimize SE in *Pinus oocarpa* by designing culture media that mimic the mineral composition of the megagametophyte have met with only limited success, yielding only modest improvements in tissue proliferation and somatic embryo maturation [130]. Furthermore, embryogenic lines derived from different ZEs exhibit varying nutritional requirements, as evidenced by differences in growth rates on identical media formulations [110].

Overall, the highly variable responses of different *Pinus* species and even genotypes within the same species to culture media and their modifications highlight the existence of species- and genotype-specific nutritional requirements that must be empirically determined for successful SE [98].

### 2.4. Growth Regulators

Plant growth regulators (PGRs) are essential in most of the five key steps of successful SE in *Pinus* species [19,47]: (i) initiation of embryogenic cultures—culturing the primary explant (usually megagametophytes or isolated immature/mature ZEs) on medium supplemented with PGRs, primarily auxin and frequently cytokinin, to induce embryogenic tissue; (ii) proliferation—maintenance and multiplication of embryogenic cultures on solid or liquid medium containing the same or reduced levels of PGRs; (iii) prematuration—transfer to PGR-free medium to inhibit proliferation and promote somatic embryo formation and early development; (iv) maturation—development of cotyledonary embryos on medium with high osmotic pressure and supplemented with abscisic acid (ABA); and (v) germination and plantlet conversion on PGR-free medium.

Plant somatic cells possess complete genetic information required to regenerate a complete, functional plant. This totipotency of plant somatic cells underlies the entire process of SE. However, for embryogenic development to be triggered, the existing gene-expression pattern in the explant must first be suppressed and subsequently reprogrammed toward an embryogenic program. One of the key mechanisms for the initial downregulation of preexisting gene expression is auxin-induced DNA hypermethylation [19,47,131,132].

Although auxin alone can sometimes trigger the earliest dedifferentiation steps, successful initiation of embryogenic cultures in *Pinus* species almost invariably requires the combined presence of an auxin and a cytokinin [19,23,39,75]. Optimal type and concentration of these PGRs are usually determined empirically or adopted from protocols established for related species. The most commonly reported PGR combinations for initiation include: 2,4-dichlorophenoxyacetic acid (2,4-D) + N^6^-benzyladenine (BA) [55,58,59,79,109,117], 1-naphthaleneacetic acid (NAA) + BA [79,92], 2,4-D + kinetin (KIN) [133]. Among these, 2,4-D in combination with BA is by far the most widely used and generally the most effective. Highest initiation frequencies are often obtained when 2,4-D and BA are applied at equimolar concentrations [59] or when BA is included at approximately one-fifth the concentration of 2,4-D [55,79]. In some protocols, a short-term pulse treatment (7–14 days) of relatively high 2,4-D followed by transfer to PGR-free or low-PGR medium has also proved successful, yielding embryogenic tissue from up to 30% of cultured ovules [99].

2,4-D remains the most widely used and effective auxin for initiating SE in conifers, likely due to its role as a strong oxidative stress inducer [20,134]. In *P. nigra*, relatively high initiation frequencies have been achieved using either NAA or 2,4-D as the only exogenous PGR [59]. Reducing PGR concentrations below standard levels can significantly improve SE initiation rates (e.g., from 16.7 to 35%) in some species [114]. In addition to auxins and cytokinins, ABA supplied during the initiation phase can enhance the induction of embryogenic tissue, possibly because early exposure mimics the natural hormonal progression in developing seeds [118]. Differences in exogenous PGR requirements among species and genotypes are largely attributed to variations in the endogenous hormonal milieu of the developing ovule, which modulates responsiveness to externally supplied growth regulators [114].

Exogenously supplied PGRs are not strictly essential for initiating SE in conifers when explants are collected at an optimal developmental stage, although they markedly increase initiation frequency [55]. Successful initiation on PGR-free media has been reported in several *Pinus* species, indicating that megagametophytes can provide sufficient endogenous hormones to trigger embryogenic culture induction. Examples include *P. nigra* [59], *P. radiata* on medium supplemented only with activated charcoal [101], *P. heldreichii* (3.3% initiation frequency when megagametophytes predominantly contained immature cleavage embryos; [92]), *P. pinaster* and *P. sylvestris* (relatively high frequency but with a 3–6 week delay in development; [135]), and *P. palustris* (a single embryogenic line from 192 explants; [58]). In some *Pinus* species, embryogenic cultures could be induced when megagametophytes were cultured on completely PGR-free medium, yet continuous proliferation of the initiated tissue still required subsequent transfer to medium containing 2,4-D and BA [117].

The proliferation medium is typically identical to the initiation medium, with white-to-translucent, glossy, and mucilaginous embryogenic tissue actively proliferating on the same 2,4-D + BA combination used for induction [53,104]. In some protocols, however, PGR concentrations are reduced during the proliferation phase: to one-tenth in *P. elliottii* [128] and stepwise to one-fifth and then one-tenth in *P. heldreichii* [92,96].

Successful maturation of SEs requires transfer of proliferating embryogenic tissue to a dedicated maturation medium. In pine species, this medium typically lacks auxins and cytokinins but is supplemented with ABA, often in combination with increased osmotic potential. Removal or strong reduction in auxin (and cytokinin) inhibits continued cleavage polyembryony, thereby allowing SEs to progress to stages that are competent to respond to ABA [39]. ABA is the most effective PGR for promoting somatic embryo maturation in conifers. Optimal ABA concentrations vary considerably among species [104] and even among cell lines of the same species; for example, when 20 embryogenic cell lines of *P. koraiensis* were tested, only ten produced cotyledonary embryos on the same maturation medium [82].

ABA is universally recognized as the key regulator of embryo maturation in both ZE and SE of gymnosperms. In developing seeds, ABA is synthesized primarily in the megagametophyte, accumulates during embryo maturation, and is subsequently transported to the developing embryo [136]. In vitro, when the megagametophyte is absent, exogenous ABA must be supplied via the culture medium [20]. ABA performs several critical functions: primarily, it prevents precocious germination of the ZEs [45,137], promotes the accumulation of storage reserves, and enhances developmental progression toward fully mature embryos. For most pine species, effective maturation in vitro occurs at ABA concentrations of 10–60 µM [8], although both lower [45,138] and higher concentrations [133,139] have been used successfully. Extremely high ABA levels, however, often reduce maturation efficiency [140].

Klimaszewska et al. [114] demonstrated that the type and concentration of PGRs applied during the initiation and proliferation phases have a pronounced effect on subsequent maturation success. High concentrations of 2,4-D and BA during proliferation consistently resulted in low yields of mature SEs. The authors proposed that residual auxin and cytokinin persisting in the embryogenic tissue interfere with early maturation events, thereby inhibiting the development of some SEs even after transfer to ABA-containing medium.

The maturation process culminates in the formation of cotyledonary SEs. These structures are capable of germination, typically achieved on PGR-free media, leading to subsequent plantlet regeneration. The optimal germination medium varies; some studies utilize a PGR-free medium supplemented with activated charcoal and maltose [59], while others incorporate growth regulators such as indole-3-butyric acid (IBA) and gibberelic acid (GA_3_) [53]. Successful plant regeneration allows for the transfer of the resulting plants to soil.

### 2.5. Organic Additives

In addition to PGRs, several organic compounds are commonly included in culture media for conifer SE. These additives serve as carbon and energy sources, osmotic agents, nitrogen supplements, or adsorbents of inhibitory compounds, and their type and concentration can markedly influence initiation, proliferation, and especially maturation success.

Sucrose is the most commonly used carbon source during both initiation and proliferation of embryogenic cultures in *Pinus* species. Typically 3% sucrose is employed [53,92], although some protocols use 2% for induction and 3% for proliferation [94]. When different carbohydrates were compared for SE induction in *P. palustris*, sucrose gave the highest frequency (4.9%), followed by maltose (3.8%) and glucose (2.4%) [58]. Embryogenic tissue has been successfully proliferated on medium containing sucrose, myo-inositol, L-glutamine, and casein hydrolysate [53]. The addition of L-glutamine, in combination with 3% or 6% sucrose, resulted in the formation of a larger number of SEs in *P. patula* [45]. Casein hydrolysate appears unnecessary for the initiation of embryogenic tissue; however, it seems required for long-term maintenance of established embryogenic cultures [58]. Supplementation of the initiation medium with myo-inositol (which also raises medium osmolality) resulted in statistically significant increases in embryogenic tissue initiation [141].

Carbohydrates also play a significant role during the maturation phase. In *P. pinaster* [142] and *P. strobus* [143], sucrose improved somatic embryo maturation when combined with high gellan gum concentrations or mannitol. Substitution of sucrose with maltose, often together with higher ABA concentrations, promoted the development of well-formed cotyledonary embryos capable of somatic seedling regeneration [104]. In some protocols, after several weeks of culture, the tissues with developing SEs were transferred to ABA-free medium with reduced maltose concentration [59]. For maturation of slash pine SEs, embryogenic tissue needs to be transferred to medium containing ABA, polyethylene glycol (PEG), and activated charcoal; PEG regulates osmotic potential, whereas activated charcoal adsorbs inhibitory compounds and residual hormones, thereby enhancing maturation [39,101,144]. Gellan gum has also been reported to promote the maturation of large numbers of SEs [141].

### 2.6. Light Requirements

SE in *Pinus* species is generally initiated and maintained in darkness, although light conditions may vary depending on the specific phase and species. For many *Pinus* species, the initiation, proliferation, and maturation phases, up to the cotyledonary stage, are successfully carried out entirely in darkness [45,58,110].

In contrast, germination and further development of SEs typically require light. This step usually involves transfer to a 16 h light/8 h dark photoperiod, which promotes greening and elongation [21,52,110]. Some protocols, however, induce germination under continuous light [94,128].

The specific requirements vary. In *P. strobus*, while the early stages occur in darkness, germination can start either in complete darkness or under red light before embryos are transferred to low-intensity white light [8]. Similarly, for *P. pinaster*, all early stages and the initial 10–15 days of germination are performed in darkness before the move to dim light [51], and in *P. oocarpa*, maturation and the initial seven days of germination are dark-phase only, followed by transfer to cool white fluorescent light [130]. *P. nigra* is distinct, as its embryogenic calli can be maintained in darkness or under a 16 h photoperiod without affecting their white, mucilaginous state [126].

## 3. Somatic Embryo Development in Conifers

Pine ovules typically contain multiple ZEs due to simple polyembryony (separate pollen grains fertilizing different egg cells, resulting in genetically distinct proembryos) and cleavage polyembryony (splitting of a single proembryo into genetically identical ones) [20,117,145]. Subsequently, only one zygotic embryo continues to develop and mature, and suppresses or eliminates the remaining embryos [47]. Arrested subordinate embryos can serve as excellent starting material for initiating SE [20,117].

SE in conifers generally follows the developmental pattern of ZE [40,126]. SEs can arise from single cells, small cell aggregates, meristematic cells within the suspensor, or through a mechanism resembling cleavage polyembryony [101]. The mode of origin—unicellular or multicellular—largely depends on the developmental stage of the explant. In more mature explants, where embryogenic competence is restricted to isolated cells, SEs typically arise from a single cell. In younger explants, where clusters of neighboring cells share similar developmental potential, groups of cells may collectively initiate embryogenesis. Regardless of their origin, all SEs follow a comparable developmental pathway, either directly from a single embryogenic cell or indirectly via a proembryogenic mass (PEM) [101].

Determining the origin of somatic embryogenic tissue in pines is particularly challenging when immature ovules (containing megagametophytes) are used as explants, primarily because multiple ZEs are already present prior to culture initiation [115], and histological examination cannot reliably distinguish small ZEs from early proliferating SEs [115,117].

Conifer embryogenic cultures typically contain numerous embryo initials, each consisting of a small cluster of 4–20 isodiametric, cytoplasm-rich cells attached to elongated, highly vacuolated suspensor (or suspensor-like) cells. These structures are embedded in a moist, mucilaginous matrix [115,117].

Throughout development, SEs maintain bipolar organization. The simplest bipolar structures comprise a single isodiametric, cytoplasm-rich embryogenic cell attached to a large, highly vacuolated cell that eventually undergoes programmed cell death [126,146]. The embryogenic cell and its derivatives first divide anticlinally, forming a linear row of densely cytoplasmic cells. Subsequent periclinal divisions produce a cylindrical embryonal mass. Cells at the proximal end of early embryonal mass become vacuolated and elongate to form the secondary suspensor, whereas distal cells remain densely cytoplasmic and constitute the embryonal region [126].

Conifer embryogenic cultures predominantly contain immature SEs that correspond to early developmental stages of ZEs, although proembryo-like structures may also occur [91]. Embryogenic cell lines exhibit considerable structural heterogeneity. Three distinct stages of PEM and their transition to early SEs have been described in conifers [82]:

- PEM I: Small clumps of densely cytoplasmic embryogenic cells attached to a single highly vacuolated suspensor cell; these form well-organized bipolar early SEs with a regularly outlined embryonal region and long vacuolated suspensor cells arranged in bundles.
- PEM II: Similar embryogenic cell clusters attached to multiple vacuolated suspensor cells; the resulting SEs are less organized, with the embryonal region consisting of a loosely aggregated mass of meristematic cells and suspensor cells lacking bundle organization.
- PEM III: Larger, more compact clumps of densely cytoplasmic embryogenic cells subtended by multiple suspensor cells; cultures are dominated by unorganized meristematic cell aggregates, with only occasional bipolar structures consisting of a few meristematic cells attached to one or two elongated vacuolated cells.

These PEM stages represent a continuum of organization in embryogenic cultures. Early SEs are distinctly polarized structures featuring a cylindrical embryonal mass and an elongated suspensor region. Only cell lines corresponding to PEM I—those containing well-organized bipolar early SEs with a clearly defined embryonal region and regularly bundled suspensor cells—reliably progress to cotyledonary SEs capable of germination and somatic seedling regeneration. In contrast, less organized lines (PEM II and PEM III), dominated by loose meristematic aggregates or irregular suspensor arrangements, rarely advance to maturation [104,147]. Accordingly, maturation treatments should be applied only after cultures have reached the well-organized PEM I stage, as premature exposure, before proper developmental advancement is reached, often inhibits further development [19].

Mature translucent SEs display a well-defined embryonal apex composed of small meristematic cells rich in dense cytoplasm, mitochondria, and starch grains. This apex is subtended by a suspensor consisting of elongated, highly vacuolated cells that contain fewer amyloplasts. The suspensor region serves as a key storage site, accumulating numerous starch granules as energy reserves, while organelles are restricted to a thin peripheral cytoplasmic layer [55]. Even under optimal maturation conditions, many embryogenic lines fail to produce cotyledonary SEs, with overall yield remaining strongly genotype-dependent [79].

## 4. Somatic Embryogenesis in *P. peuce* and *P. heldreichii*

### 4.1. Initiation of Embryogenic Tissue

To overcome seed dormancy and accelerate propagation, particularly in endangered conifers or species with extremely low natural germination rates, researchers increasingly substitute the megagametophyte with optimized nutrient media, PGRs, and controlled physical conditions. Studies investigating the nutritional, hormonal, and physical requirements of ZEs have identified culture conditions that significantly enhance germination and support SE in *P. heldreichii* [148] and *P. peuce* [149]. These optimized protocols have subsequently been applied to induce embryogenic tissues, thereby shortening the reproductive cycle and facilitating rapid propagation and conservation efforts.

SE induction has initially been attempted in *P. heldreichii* and *P. peuce* using three types of explants: (1) mature ZEs, (2) isolated immature ZEs, and (3) immature ovules containing the intact megagametophyte. Explants of types (1) and (2) consistently failed to produce embryogenic tissue in either species. For mature ZEs, isolated from mature seeds of both Bosnian pine and Macedonian pine, a range of basal media (GDS, MS, LP and DCR) supplemented with various combinations and concentrations of auxins (2,4-D or NAA) and cytokinin (BA) were tested. None of these treatments induced embryogenic cultures; instead, mature ZEs either germinated precociously or produced only non-embryogenic callus in *P. heldreichii* [148] and *P. peuce*. While high-ammonium media such as LP and MS, or DCR (containing substantial ammonium levels) are frequently used for conifer somatic embryogenesis, ZEs of the tested pines exhibited poor survival on these media, with only sporadic germination or formation of non-embryogenic callus. In contrast, GDS medium, with its lower ammonium content (approximately half that of DCR), proved markedly superior for both pines studied. This suggests that reduced ammonium may alleviate potential toxicity or imbalance in nitrogen assimilation, providing valuable guidance for optimizing protocols in ammonium-sensitive pine species. Similarly, isolated immature ZEs at the cotyledonary stage were cultured on GDS medium, GDS with nitrogen compounds reduced by half (GDS1), or GDS with nitrogen compounds completely omitted (GDS2), again all supplemented with different auxin–cytokinin combinations. Despite these extensive modifications, no embryogenic cultures were obtained in either *P. heldreichii* or *P. peuce*. The resulting callus was exclusively non-embryogenic, and no somatic embryo-like structures were observed. Microscopic examination revealed loosely organized isodiametric cells of variable size that divided in a disorganized fashion, with no PEMs or early somatic embryo-like structures observed.

In contrast, embryogenic tissue was successfully initiated only from whole megagametophytes containing immature ZEs (type 3 explant) in both *P. peuce* and *P. heldreichii* [92,96]. Initiation occurred when the enclosed ZEs were at one of four developmental stages: (i) immature cleavage (cleavage polyembryony), (ii) immature cleavage–precotyledonary, (iii) precotyledonary–early cotyledonary, or (iv) early cotyledonary. The optimal period for cone collection extended from the fourth week of June to the third week of July (approximately 4–10 weeks after fertilization), depending on the seed family sampled. At that time, isolated megagametophytes appeared translucent (late June/early July collections) to slightly opaque or whitish (second and third weeks of July). The effect of zygotic embryo developmental stage on embryogenic tissue initiation frequency in *P. peuce* is presented in Table 2.

The data presented in Table 2 encompass experiments conducted over a decade (2003–2013), a period during which notable advances in conifer somatic embryogenesis occurred. However, the key findings regarding media performance remain representative and valid under current technical frameworks. This superiority stems from the physiological ammonium sensitivity of the tested pine species, a trait that is inherent and consistent across years, independent of subsequent protocol improvements focused primarily on proliferation, maturation, or germination stages.

**Figure 1:**
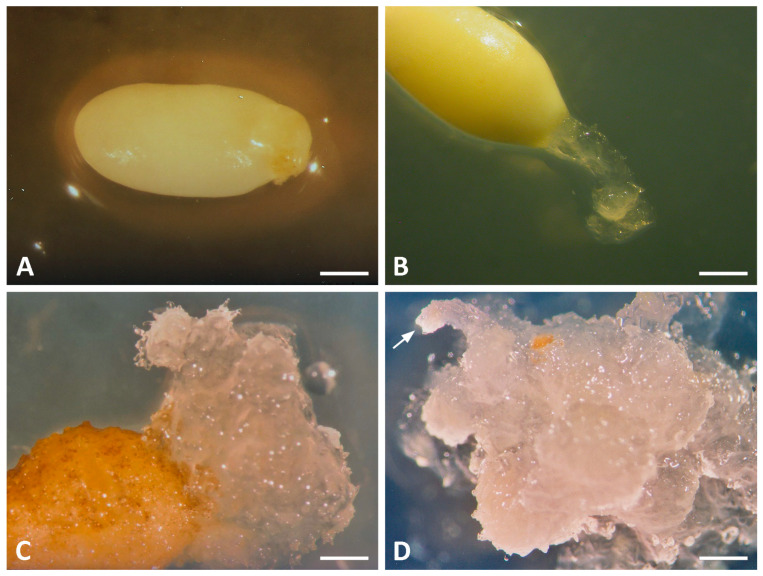
Stages of somatic embryogenesis in *Pinus peuce*. (**A**) Megagametophyte after 3 days on induction medium. (**B**) Protrusion of translucent, mucilaginous embryogenic tissue from the micropylar end, spreading across the medium surface in a strip. (**C**) Proliferation of the embryogenic tissue. (**D**) Developing embryos during early maturation phase on medium with ABA and maltose. Arrow indicates the embryo head embedded in a mucilaginous matrix. Scale bars: 1 mm (**A**–**C**); 4 mm (**D**).

Megagametophytes were initially cultured in darkness on GDS medium [14] containing 2 mg L^−1^ of either 2,4-D or NAA and 0.5 mg L^−1^ BA to initiate SE (5-day primary treatment) (Figure 1A). In addition to the standard GDS medium, variants with nitrogen salts reduced by half (GDS1) or completely omitted (GDS2) were tested (Table 2), as were further modifications (in 2011) involving the complete omission of individual macronutrients (nitrogen, chloride, magnesium, dihydrogen phosphate, or hydrogen phosphate), each supplemented with 2,4-D (2 mg L^−1^) and BA (0.5 mg L^−1^) and using 30 explants per treatment. After 5 days, explants were transferred to the same medium but with PGR concentrations decreased to one-fifth of the initial levels (secondary treatment). Subculturing was performed at 2-week intervals. During the secondary treatment, embryogenic tissue protruded from the micropylar end of the megagametophyte (Figure 1B). This led to the establishment of typical conifer embryogenic tissue in *P. heldreichii* [92,96] and *P. peuce* (Figure 1C,D).

When megagametophytes containing ZEs at the cleavage polyembryony stage were cultured, embryogenic tissue was initiated from 3.3 to 16.7% of explants across all media tested except two (Table 2). Higher average initiation frequencies were obtained on media supplemented with 2,4-D and BA (8.3%) compared to NAA and BA (6.3%). In the 2011 experiment, involving the complete omission of individual macronutrients from the GDS medium supplemented with 2,4-D and BA, initiation completely failed when nitrogen, chloride or magnesium were omitted. Similarly, megagametophytes containing ZEs at the immature cleavage–precotyledonary stage yielded embryogenic tissue initiation in 3.3–6.7% (Table 2). In contrast, explants with embryos at the precotyledonary to early cotyledonary stage rarely produced embryogenic tissue, with initiation observed only sporadically—three cases in *P. peuce* (Table 2) and a single case in *P. heldreichii* [69]. Comparable low initiation frequencies (2.6% and 0.99% in a later study) from intact megagametophytes containing precotyledonary ZEs have been reported in *P. patula* [45,55]. Megagametophytes containing more advanced cotyledonary embryos typically formed only non-embryogenic callus characterized by loosely arranged, elongated, and vacuolated cells. This type of callus was not enveloped in mucilaginous gel, and microscopic examination revealed no SEs. As zygotic embryo development within the megagametophytes progressed, initiation frequencies declined sharply, falling from an initial 16.7% to 0% in both *P. heldreichii* [92] and *P. peuce* (Table 2), irrespective of the culture medium used. Similar results were obtained in *P. nigra*, where initiation rates dropped from 8% to 0–1% [104]. The culmination of embryogenic induction from immature explants thus appears to coincide with the termination of cleavage polyembryony in the zygotic embryo, after which initiation success rapidly decreases [45]. Cleavage polyembryony has been identified as the optimal developmental stage for initiation of SE in *P. patula* [45,55], corresponding to zygotic embryo lengths of 0.25–1.35 mm.

### 4.2. Proliferation and Maintenance

Once initiation had occurred, after four weeks of secondary treatment, megagametophytes of *P. peuce* and *P. heldreichii* [92] with attached mucilaginous tissue were transferred to an embryogenic culture proliferation and maintenance medium in which PGR concentrations were reduced to one-tenth of the original levels. The cultures were then subcultured every two weeks and maintained in the dark. A stepwise reduction in PGR concentrations (first to one-fifth and then to one-tenth of the original concentration) has also proved effective in *P. elliottii* [128]. In conifers generally, sustained proliferation of embryogenic tissue—regardless of explant type—requires transfer to a medium with reduced PGR levels [100].

**Figure 2:**
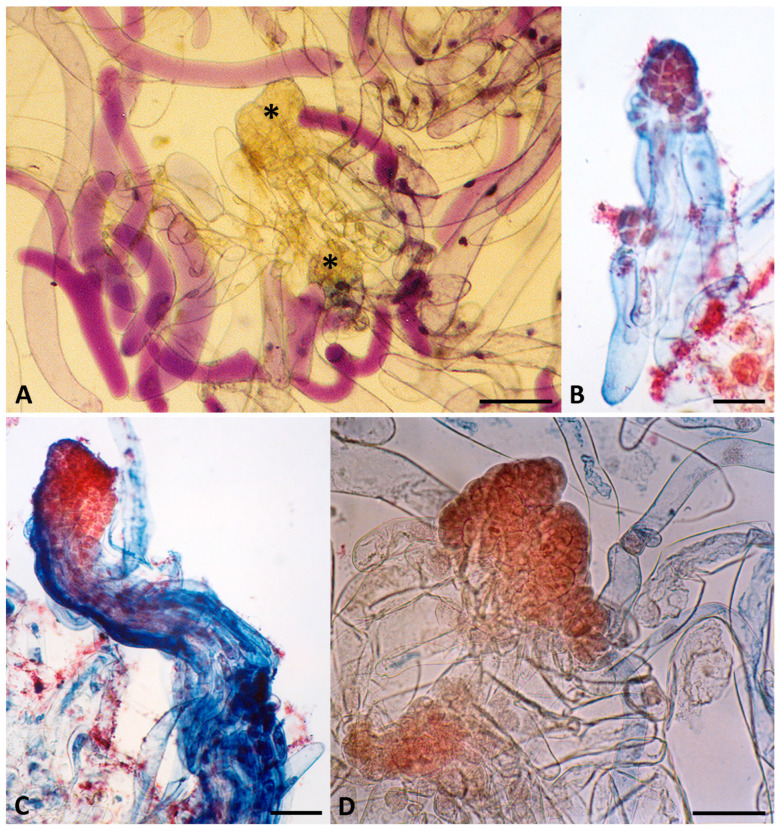
Histological analysis of proliferating Macedonian pine embryogenic tissue. (**A**) Embryogenic tissue containing early SEs (asterisks). (**B**) Early somatic embryo with developing, densely cytoplasmic embryo head (stained red with 2% acetocarmine) and elongated suspensor cells (stained blue with 0.05% Evans blue). (**C**) More advanced developmental stage of somatic embryo, with a clearly differentiated embryo head subtended by elongated suspensor. (**D**) Cleavage polyembryony. Scale bars: 200 μm.

In *P. peuce*, the emerging embryogenic tissue was mucilaginous and translucent (Figure 1). Histological analyses revealed three tissue types during initiation: embryogenic, non-embryogenic, and mixtures of both, as observed in *P. patula* [55]. Non-embryogenic tissue, originating probably from the surface of the female gametophyte, consisted of small, compact, spherical cells and never gave rise to embryogenic structures. This tissue often overgrew and rapidly smothered the shorter-lived embryogenic tissue. Embryogenic tissue consisted of early SEs at various developmental stages, clumps of disorganized dividing vegetative cells, and elongated suspensor cells (Figure 2A). Following an initial period of slow growth, embryogenic tissue mass started to proliferate rapidly, reaching approximately 5 mm in diameter within a week, at which point it should be separated from the megagametophyte. Embryogenic tissue of *P. peuce* exhibited strong proliferative capacity, rapidly producing early SEs at various developmental stages (Figure 2B,C). Early embryonal mass comprised densely cytoplasmic cells with prominent, centrally located nuclei, attached to a long, highly coiled suspensor. Suspensor cells were markedly enlarged, highly vacuolated, and contained only sparse peripheral cytoplasm with conspicuous nuclei. Continued cell divisions in both regions gave rise to a distinct embryonal head and a multilayered suspensor, as observed in Macedonian pine (Figure 2D), Bosnian pine [92], and many other *Pinus* species [58]. Subculture onto fresh medium promoted further differentiation into more advanced SEs.

Once established and separated from the explant, embryogenic cultures were maintained by regular subculture every two weeks. During long-term maintenance, the regeneration capacity of embryogenic cell lines gradually declined. While some lines of Macedonian pine retained proliferative and regeneration-competent status for more than one year—similar to observations in Bosnian pine [92]—others lost this regeneration ability after extended culture periods. This variability prompted the annual initiation of new embryogenic cell lines. Somatic embryo production capacity declined rapidly during proliferation [92], representing one of the major obstacles to commercial application of SE techniques [79]. Prolonged subculturing on fresh medium is known to reduce maturation capacity, particularly in *Pinus* species [150]. This decline can be mitigated by transferring cultures to a maltose-based maturation medium without PGRs or by performing weekly subcultures [151].

### 4.3. Prematuration

In *P. peuce*, new SEs continuously form on proliferation media supplemented with 2,4-D or NAA in combination with BA. Another critical step in the SE process is halting further proliferation to allow the embryos to enter the maturation phase. Therefore, to promote further development of the SEs it was necessary to transfer the embryogenic cultures to a medium with reduced PGR concentrations, followed by their complete omission [52], or to a PGR-free medium [8] or a medium supplemented with activated charcoal [128].

Applying maturation treatments prematurely—before SEs have reached the appropriate developmental stage—can result in poor embryo quality, abnormal morphology, or insufficient accumulation of storage reserves. As noted by von Arnold et al. [19], embryos should only be exposed to maturation conditions (typically involving ABA) once they have advanced sufficiently in development. Arrested development at this stage often leads to low maturation yields, precocious (premature) germination, or embryos lacking the vigor for successful germination and acclimatization.

### 4.4. Maturation and Somatic Embryo Germination

To stimulate maturation in embryogenic cultures of different *Pinus* species, ABA is typically required at concentrations of 10–50 µM. The optimal ABA concentration varies among species, and treatment is effective only when the embryogenic tissue has reached a responsive developmental stage—a key determining factor [128]. In general, a one-month treatment is optimal, although prolonged exposure to ABA increases the number of mature embryos but can have negative after-effects on subsequent plant growth [19]. Maturation protocols commonly employ ABA to promote storage compound accumulation and to suppress secondary embryogenesis or inhibit precocious germination [19]. Genotype-specific responses and prolonged exposure to proliferation hormones can disrupt this transition, highlighting the need for precise timing and medium optimization. In *P. nigra*, for example, low ABA concentrations resulted in limited somatic embryo development and no seedling regeneration, whereas high concentrations (95 µM) combined with maltose (3%, 6%, or 9%) produced well-developed SEs capable of regenerating seedlings [104].

Specific protocols have been tested in Balkan pines. For embryo maturation in *P. peuce* and *P. heldreichii* [92], embryogenic tissue was first transferred to a PGR-free medium to inhibit cleavage polyembryony, then placed on a filter paper disk on the surface of one of several tested maturation media: PGR–free; ABA (0.3, 3, 6, 9, or 12 mg L^−1^, equivalent to approximately 1–45 µM); PGR-free for 14 days followed by ABA (0.3–12 mg L^−1^); sucrose (5%) with ABA (0.3–12 mg L^−1^); maltose (3% or 5%); *p*-chlorophenoxyisobutyric acid (PCIB, 0.22 mg L^−1^); or PCIB (0.22 mg L^−1^) with ABA (6 mg L^−1^). To facilitate partial desiccation of the embryogenic mass, the filter paper disk was exposed to laminar airflow for 2 h at room temperature, after which the Petri dishes were sealed and maintained under a 16 h light/8 h dark photoperiod, with monthly subcultures onto fresh medium. A similar but more prolonged partial desiccation approach—exposing filter paper-supported tissue to laminar airflow for 6 h at room temperature, to achieve approximately 23% water loss—has been successfully applied in *P. pinea* [52].

Cultivation on ABA-containing medium promoted the initial stages of somatic embryo maturation, with the highest responsiveness achieved at 12 mg L^−1^ ABA combined with an increased sucrose concentration. Single embryos increased slightly in size, and firm, opaque to yellowish embryo heads became noticeable within the mucilaginous matrix, with tens of embryos observed per culture after one week. Over the next 3–4 weeks, most embryogenic cultures of *P. heldreichii* turned creamy yellow; some SEs became brownish and failed to complete maturation [92]. In *P. peuce*, only a few embryos remained vigorous. Immature SEs of *P. peuce* were manually isolated from proliferating tissue and placed directly on the surface of the maturation medium (without filter paper) to complete their development. However, addition of maltose, the anti-auxin PCIB (alone or in combination with ABA), or ABA following a 2-week PGR-free pretreatment to the maturation medium failed to induce maturation in either species.

The maturation process culminates in the formation of cotyledonary SEs capable of germination, typically on hormone-free media, followed by regeneration into plantlets. Germination media for *Pinus* species usually have the same basal composition as the initiation and proliferation media but lack PGRs. The addition of activated charcoal (AC) is common in many protocols and has been reported to significantly improve both maturation and germination rates. For example, in *P. elliottii*, incorporating AC during germination resulted in a 63% germination rate and an 85% survival rate of seedlings derived from SEs [27]. Protocols for other species, such as *P. nigra* [147] and *P. radiata* [21], also routinely include AC. AC plays an essential role through its capacity to adsorb phenolics and residual PGRs, although its effect—stimulatory or inhibitory—can vary depending on the species and tissue [152].

Nevertheless, successful germination without AC has been achieved in certain species, including *P. strobus* [114] and *P. pinea* [52]. However, SEs of *P. peuce* cultured on PGR-free medium under a 16 h light/8 h dark photoperiod failed to germinate. Overall, these variations highlight the need for species-specific optimizations in germination protocols to maximize the success of SE in *Pinus spp*.

While the protocols described above rely on empirical optimization, recent molecular studies in pines and other conifers provide deeper insights into the regulatory networks that could guide future refinements for recalcitrant species like *P. peuce* and *P. heldreichii*. Recent advances in the molecular regulation of SE in pines and conifers highlight the central role of ABA-dependent mechanisms that promote maturation, storage reserve accumulation, suppression of precocious germination, and stress adaptation—processes essential for successful SE [90,153,154]. Transcriptomic and proteomic analyses in *P*. *pinaster* under reduced water availability or temperature variations demonstrate early activation of protective pathways, differential expression of stress-related genes and epigenetic regulators, redox homeostasis components, and carbohydrate metabolism adjustments, mediated by ABA to facilitate maturation, embryo quality, and long-term plant resilience [153,155,156].

In conifers, including several *Pinus* species, homologues of angiosperm LAFL network genes (*LEC1*, *ABI3*, *FUS3*, *LEC2*), serving as a central regulatory hub for totipotency, embryo maturation, and integration of hormonal signals, are expressed during SE [154]. However, in silico analyses suggest that LEC2—one of the key regulators of SE initiation in angiosperms—may be absent in some conifer species [157]. This potential absence could contribute to some of the characteristic challenges in conifer SE (such as variable initiation rates and genotype sensitivity), although alternative compensatory mechanisms (e.g., enhanced LEC1 or ABI3/FUS3 roles in auxin/ABA integration) likely exist. While no species-specific molecular data are currently available for *P. heldreichii* and *P. peuce*, these conserved mechanisms from other pines and conifers likely apply. This underscores the importance of precise ABA timing, osmotic stress application, and targeted modulation of conserved pathways to overcome maturation and germination bottlenecks in recalcitrant Balkan pines, paving the way for more efficient SE protocols in the future.

## 5. Conclusions

The synthesis presented in this review confirms that SE is a technologically viable route for the mass clonal propagation of *Pinus* species, offering significant advantages in genetic uniformity and circumventing the rooting bottleneck associated with organogenesis. However, the transition of SE from laboratory success to operational, industrial-scale deployment remains hindered by a persistent suite of technical hurdles, notably low initiation frequencies and strong genotype dependence across the genus.

Our analysis, specifically focusing on the critical Balkan species *P. peuce* and *P. heldreichii*, underscores the extreme sensitivity of the induction phase. For these valuable species, SE initiation was strictly limited to the culture of whole megagametophytes containing immature ZEs. Crucially, the process failed when using either mature ZEs or isolated immature embryos, highlighting a dependence on the nutritional and hormonal cues provided by the surrounding megagametophyte tissue during a narrow 4–10-week collection period. These species-specific insights into the stringent explant, developmental-stage, and culture medium requirements are essential.

By identifying these key bottlenecks, this work provides a necessary framework for future efforts to develop robust, standardized, and high-yielding SE protocols, thereby securing the long-term conservation and sustainable use of these ecologically and economically important pines in future afforestation programs.

## 6. Future Directions

The path toward the operational deployment of somatic embryogenesis for *P. peuce* and *P. heldreichii*, and the *Pinus* genus more broadly, requires concentrated efforts to overcome the developmental and genotypic hurdles identified in this review. Future research should prioritize three key areas:

Explant reprogramming and optimization is a major future direction, focusing on the development of robust protocols that permit SE initiation from non-juvenile tissues, such as mature ZEs or vegetative explants from elite, field-tested mature trees. This would remove the current bottleneck of limited seasonal availability and the reliance on whole megagametophytes.

Physiological and molecular characterization is necessary; to increase the low and highly variable initiation rates, future work must focus on elucidating the underlying molecular and physiological mechanisms that govern the transition to embryogenic competence. Techniques such as transcriptomics and metabolomics can help define the optimal endogenous hormonal and nutrient status required for successful SE induction, moving beyond the current empirical, trial-and-error optimization.

Finally, maturation and field-deployment efficiency must be addressed; to realize the industrial potential of SE, subsequent research must focus on optimizing the later developmental stages, including maturation, germination, and acclimatization protocols. The ultimate goal is to generate high-quality, phenotypically stable somatic seedlings with enhanced survival rates that can be successfully deployed in large-scale reforestation and forest conservation programs.

## Figures and Tables

**Table 1:** Composition of selected basal media: MS (Murashige and Skoog [120]), GDS (Gresshoff and Doy [121] modified by Sommer et al. [14]), LP (von Arnold and Eriksson [122]), SH (Schenk and Hildebrandt [123]), and DCR (Gupta and Durzan [124]).

Constituents	MS	GDS	LP	SH	DCR
**Macronutrients (mg L^−1^)**					
NH_4_NO_3_	1650	-	1200	-	400
KNO_3_	1900	1000	1900	2500	340
CaCl_2_ × 2H_2_O	440	150	180	148	85
MgSO_4_ × 7H_2_O	370	250	370	370	370
KH_2_PO_4_	170	-	340	136	170
(NH_4_)_2_SO_4_	-	200	-	134	-
KCl	-	300	-	-	-
NaH_2_PO_4_ × H_2_O	-	90	-	-	-
Na_2_HPO_4_	-	30	-	-	-
Ca(NO_3_)_2_ × 4H_2_O	-	-	-	-	556
**Micronutrients (mg L^−1^)**					
Mn SO_4_ × 4H_2_O	22.3	-	22.0	-	2.23
Zn SO_4_ × 7H_2_O	8.6	5.4	-	8.6	0.86
H_3_BO_3_	6.2	3.0	0.63	6.2	0.62
KI	0.83	0.75	0.75	0.83	0.083
NaMoO_4_ × 2H_2_O	0.25	0.25	0.025	0.25	0.025
CuSO_4_ × 5H_2_O	0.025	0.25	0.0025	0.025	0.025
CoCl2 × 6H_2_O	0.025	0.25	0.0025	0.02	0.0025
FeSO_4_ × 7H_2_O	27.8	27.8	14.0	27.85	27.8
Na_2_EDTA	37.3	37.3	24.0	37.300	37.3
MnSO_4_ × H_2_O	-	10.0	-	22.300	-
NiCl_2_	-	-	-	-	0.0025
**Organic additives (mg L^−1^)**					
Thiamine·HCl	0.1	1.0	5.0	0.01	0.1
Pyridoxine·HCl	0.5	0.1	1.0	-	0.05
Nicotinic acid	0.5	0.1	2.0	-	0.05
Glycine	2.0	-	-	-	0.2
**Organic additives (g L^−1^)**					
myo-Inositol	0.1	0.01	0.1	0.1	0.1
Sucrose	30.0	30.0	30.0	30.0	30.0
D-Glucose	-	-	0.18	-	-
D-Xylose	-	-	0.15	-	-
L-Arabinose	-	-	0.15	-	-
Casein hydrolysate	**-**	0.5	**-**	-	0.5
L-Glutamine	**-**	0.05	**-**	-	0.05
Agar	7.0	7.0	7.0	7.0	7.0

**Table 2:** Effect of developmental stage of *Pinus peuce* zygotic embryos (determined by cone collection date) on the percentage of explants initiating embryogenic tissue. All cones were collected on Mučanj Mountain, Serbia. For each collection date, the induction period on initiation medium lasted 5 days.

Medium ^1^	Plant Growth Regulators (mg L^−1^) ^2^	Cone Collection Date			
		Last Week of June	1st Week of July	2nd Week of July	3rd Week of July
		Developmental Stage			
		Immature Cleavage	Immature Cleavage–Precotyledonary	Precotyledonary–Early Cotyledonary	Early Cotyledonary
		Initiation Frequency (%) ^3^			
		**2003**			
GDS	2,4-D (2), BA (0.5)	3.3	-	-	-
GDS	NAA (2), BA (0.5)	6.7	-	-	-
GDS1	2,4-D (2), BA (0.5)	0	-	-	-
GDS1	NAA (2), BA (0.5)	3.3	-	-	-
GDS2	2,4-D (2), BA (0.5)	10.0	-	-	-
GDS2	NAA (2), BA (0.5)	3.3	-	-	-
		**2006**			
GDS	2,4-D (2), BA (0.5)	3.3	0	0	0
GDS	NAA (2), BA (0.5)	6.7	6.7	0	0
GDS1	2,4-D (2), BA (0.5)	16.7	6.7	3.3	0
GDS1	NAA (2), BA (0.5)	6.7	0	0	0
GDS2	2,4-D (2), BA (0.5)	10.0	0	0	0
GDS2	NAA (2), BA (0.5)	3.3	0	0	0
		**2007**			
GDS	2,4-D (2), BA (0.5)	6.7	3.3	0	0
GDS	NAA (2), BA (0.5)	6.7	6.7	3.3	0
GDS1	2,4-D (2), BA (0.5)	16.7	3.3	3.3	0
GDS1	NAA (2), BA (0.5)	6.7	6.7	0	0
GDS2	2,4-D (2), BA (0.5)	6.7	0	0	0
GDS2	NAA (2), BA (0.5)	3.3	0	0	0
		**2009**			
GDS	2,4-D (2), BA (0.5)	6.7	3.3	-	-
GDS	NAA (2), BA (0.5)	16.7	6.7	-	-
GDS1	2,4-D (2), BA (0.5)	16.7	3.3	-	-
GDS1	NAA (2), BA (0.5)	3.3	3.3	-	-
GDS2	2,4-D (2), BA (0.5)	3.3	3.3	-	-
GDS2	NAA (2), BA (0.5)	0	0	-	-
		**2010**			
GDS	2,4-D (2), BA (0.5)	3.3	3.3	-	-
GDS	NAA (2), BA (0.5)	3.3	3.3	-	-
GDS1	2,4-D (2), BA (0.5)	16.7	3.3	-	-
GDS1	NAA (2), BA (0.5)	6.6	0	-	-
GDS2	2,4-D (2), BA (0.5)	3.3	0	-	-
GDS2	NAA (2), BA (0.5)	3.3	3.3	-	-
		**2011**			
GDS	2,4-D (2), BA (0.5)	3.3	3.3	0	-
GDS	NAA (2), BA (0.5)	3.3	3.3	0	-
GDS	PGR-free	6.7	6.7	0	-
GDS1	2,4-D (2), BA (0.5)	16.7	6.7	0	-
GDS1	NAA (2), BA (0.5)	10.0	0	0	-
GDS2	2,4-D (2), BA (0.5)	10.0	0	0	-
GDS2	NAA (2), BA (0.5)	6.7	3.3	0	-
		**2013**			
GDS	2,4-D (2), BA (0.5)	3.3	3.3	-	-
GDS	NAA (2), BA (0.5)	10.0	3.3	-	-
GDS1	2,4-D (2), BA (0.5)	10.0	0	-	-
GDS1	NAA (2), BA (0.5)	6.7	0	-	-
GDS2	2,4-D (2), BA (0.5)	10.0	0	-	-
GDS2	NAA (2), BA (0.5)	3.3	0	-	-

^1^ GDS—GD medium [121] modified by Sommer et al. [14]; GDS1—GDS with nitrogen salts reduced by half; GDS2—GDS with nitrogen salts completely omitted. ^2^ Plant growth regulators (PGRs) used: 2,4-D (2,4-dichlorophenoxyacetic acid), BA (N^6^-benzyladenine), NAA (1-naphthaleneacetic acid). ^3^ The value represented by the hyphen (-) denotes missing data (the specific explant was not included in the experiment for the designated year or treatment). A zero (0) indicates a negative result (the explant was utilized, but the percentage of embryogenic tissue formation was 0%).

## Data Availability

The original contributions presented in the study are included in the article, further inquiries can be directed to the corresponding author.

## Abbreviations

The following abbreviations are used in this manuscript:

SE	Somatic Embryogenesis
SEs	Somatic Embryos
ZE	Zygotic Embryogenesis
ZEs	Zygotic Embryos
PEM	Proembryogenic Mass
GD	Gresshoff and Doy [121]
GDS	GD modified by Sommer et al. [14]
PGR	Plant Growth Regulator
2,4-D	2,4-Dichlorophenoxyacetic Acid
NAA	1-Naphthaleneacetic Acid
BA	N^6^-Benzyladenine
KIN	N^6^-Furfuryladenine
ABA	Abscisic Acid
PCIB	*p*-Chlorophenoxyisobutyric Acid
